# Drainage

**Published:** 1884-05

**Authors:** 


### HALL'S JOURNAL OF HEALTH

AND

MISCELLANY.

MONTHLY.

E. H. GIBBS, A. M., M. D., EDITOR.

FOUNDED IN 1854.

### DRAINAGE

THERE is a common mistake regarding house drains—that they are made too large. It is incorrect to suppose that a very large drain is safer than one of moderate size, because the smaller the drain the more concentrated the flow, and the more thorough the flushing when larger amounts of water than usual are passed through it; as on washing days. If the liquid drainage is received directly from the kitchen without any provision for stopping grease, a twelve-inch drain will soon become partly filled with a greasy sediment; and the water-way will assume a broad, flat form, over which the flow will spread itself and become too thin and too slow to ensure the proper scouring effect.

A much smaller pipe would have this deposit of grease confined to a narrow channel, and the whole of a copious flow, being concentrated on it, would have a much better chance to cut it away and remove it. It may be taken as a rule that no private house, no matter how large, can need for its drainage, a pipe larger than four inches in diameter. Neither would it ordinarily be prudent to use a smaller pipe than this, for any house.

The smaller the amount of drainage a pipe is to carry, the more perfect must be its construction; for, a deposit of offensive matter is much more easily retained in a drain which is little used than in one which is often flushed with large quantities of liquid. Angles and curves must, if possible, be avoided in the drain; for, besides affording lodgment for the sediment, they diminish the force of the stream, on account of the increased friction they offer to it. In a straight drain, moreover, it is often easy to remove any obstruction, by means of a wire brush thrust into the end of the pipe and pushed on through, like a chimney sweeping-brush. The fall or inclination of the drain need not be great, but must be uniform, or obstructions will almost surely result. The best material for drain pipes is iron; but if the expense is considered too great the next best thing is glazed earthenware. In any case the joints must be water-tight. It is customary to lay the pipes loosely, and fill in the joints with clay; this plan is liable to result seriously for the health of the persons using the drain. The joints should be cemented together in such a way that a large mass of cement surrounds the joint. The brick, stone, and cement drains are dangerous. The importance of exercising the utmost care in building drains will be felt when it is known that the greater number of cases of diptheria and typhoid fever may be traced to defective drainage, as a cause.

